# Nanoscale-Targeted Patch-Clamp Recordings of Functional Presynaptic Ion Channels

**DOI:** 10.1016/j.neuron.2013.07.012

**Published:** 2013-09-18

**Authors:** Pavel Novak, Julia Gorelik, Umesh Vivekananda, Andrew I. Shevchuk, Yaroslav S. Ermolyuk, Russell J. Bailey, Andrew J. Bushby, Guy W.J. Moss, Dmitri A. Rusakov, David Klenerman, Dimitri M. Kullmann, Kirill E. Volynski, Yuri E. Korchev

**Affiliations:** 1 Department of Medicine, Imperial College London, London, W12 0NN, UK; 2 School of Engineering and Materials Science, Queen Mary, University of London, London E1 4NS, UK; 3 Department of Cardiac Medicine, National Heart and Lung Institute, Imperial College London W12 0NN, UK; 4 UCL Institute of Neurology, University College London, London WC1N 3BG, UK; 5 Institute for Life Sciences, University of Southampton, Southampton SO17 1BJ, UK; 6 Department of Neuroscience Physiology and Pharmacology, University College London, London WC1E 6BT, UK; 7 Centre for Mathematics and Physics in the Life Sciences and Experimental Biology, University College London, London WC1E 6BT, UK; 8 Department of Chemistry, Cambridge University, Cambridge CB2 1EW, UK

## Abstract

Direct electrical access to presynaptic ion channels has hitherto been limited to large specialized terminals such as the calyx of Held or hippocampal mossy fiber bouton. The electrophysiology and ion-channel complement of far more abundant small synaptic terminals (≤1 μm) remain poorly understood. Here we report a method based on superresolution scanning ion conductance imaging of small synapses in culture at approximately 100–150 nm 3D resolution, which allows presynaptic patch-clamp recordings in all four configurations (cell-attached, inside-out, outside-out, and whole-cell). Using this technique, we report presynaptic recordings of K^+^, Na^+^, Cl^−^, and Ca^2+^ channels. This semiautomated approach allows direct investigation of the distribution and properties of presynaptic ion channels at small central synapses.

**Video Abstract:**

## Highlights

• Topographic imaging of live synaptic boutons at nanoscale resolution
• Cell-attached patch-clamp recordings of ion channels in small central synapses
• Whole-cell small presynaptic bouton recordings

## Teaser

Novak and colleagues have developed a method for nanoscale-targeted patch-clamp presynaptic recordings in submicrometer central synapses identified using superresolution scanning ion conductance microscopy. This semiautomated approach opens a new window on the physiology of small presynaptic terminals.

## Introduction

Current knowledge of neurotransmitter release mechanisms relies mainly on studies of large synapses, such as the calyx of Held or hippocampal mossy fiber bouton (Bischofberger et al., 2006; Schneggenburger and Forsythe, 2006), which can be patch clamped to control the presynaptic membrane potential and to manipulate or measure Ca^2+^ concentrations. However, the majority of central synapses are too small (∼1 μm scale) to permit similar approaches. As a result, although recent years have witnessed substantial progress in identifying the molecules involved in activity-dependent exo- and endocytosis at such synapses (Rizo and Rosenmund, 2008; Südhof and Rothman, 2009), a quantitative understanding of ion channel properties in small presynaptic boutons remains poorly understood (Debanne et al., 2011).

The conventional patch-clamp technique relies on diffraction-limited optical microscopy to navigate a glass pipette to the target structure. In practice, this imposes a lower limit on the size of the subcellular compartment that can be targeted for recording. Consequently, even the smallest cellular structures successfully targeted using differential interference contrast (DIC) optics, such as hippocampal mossy fiber boutons (∼2–5 μm diameter) (Bischofberger et al., 2006; Ruiz et al., 2010) or axonal blebs (∼4–6 μm) (Shu et al., 2006), are an order of magnitude larger than the optical diffraction limit (∼200 nm). Recordings from narrow axons have recently been obtained using pipettes coated with fluorescently conjugated albumin; however, this method only allows cell-attached recordings of action-potential (AP) waveforms (Sasaki et al., 2012).

Here we describe a semiautomated approach that allows precise targeted recordings from small synaptic terminals in cultured hippocampal neurons in all four configurations of the patch-clamp method (cell-attached, inside-out, whole-cell, and outside-out). The technique is based on imaging structures with superresolution hopping probe ion conductance microscopy (HPICM, a variant of scanning ion conductance microscopy [SICM] [Novak et al., 2009]), followed by patch-clamp recordings from the identified structures using the same scanning nanopipette. We report the first, to our knowledge, direct ion-channel recordings from small (∼1 μm) en passant axonal varicosities. This robust semiautomated method can be used even by inexperienced electrophysiologists and therefore opens a window on the nanoscale physiology of small presynaptic terminals.

## Results

### 3D Imaging of Active Synaptic Boutons at Nanoscale Resolution

In order to identify live synaptic boutons in the complex network of neuronal cultures, we combined HPICM with fluorescence imaging of amphiphilic FM dyes, which label recycling synaptic vesicles (Gaffield and Betz, 2006). HPICM and other variants of SICM rely on an electrolyte-filled glass nanopipette sensing the distance to the cell surface via changes in the pipette current in response to a constant command voltage, with a resolution determined by the nanopipette size (∼1.5 times the inner pipette tip diameter [Rheinlaender and Schaffer, 2009]). This method can reproduce the 3D topography of live cells in culture at nanoscale resolution (down to 20 nm) (Korchev et al., 1997; Novak et al., 2009) and can be combined with subsequent single-channel patch-clamp recordings from specific locations using the same nanopipette (“smart patch clamp”) (Gorelik et al., 2002a; Gu et al., 2002).

**Figure 1:**
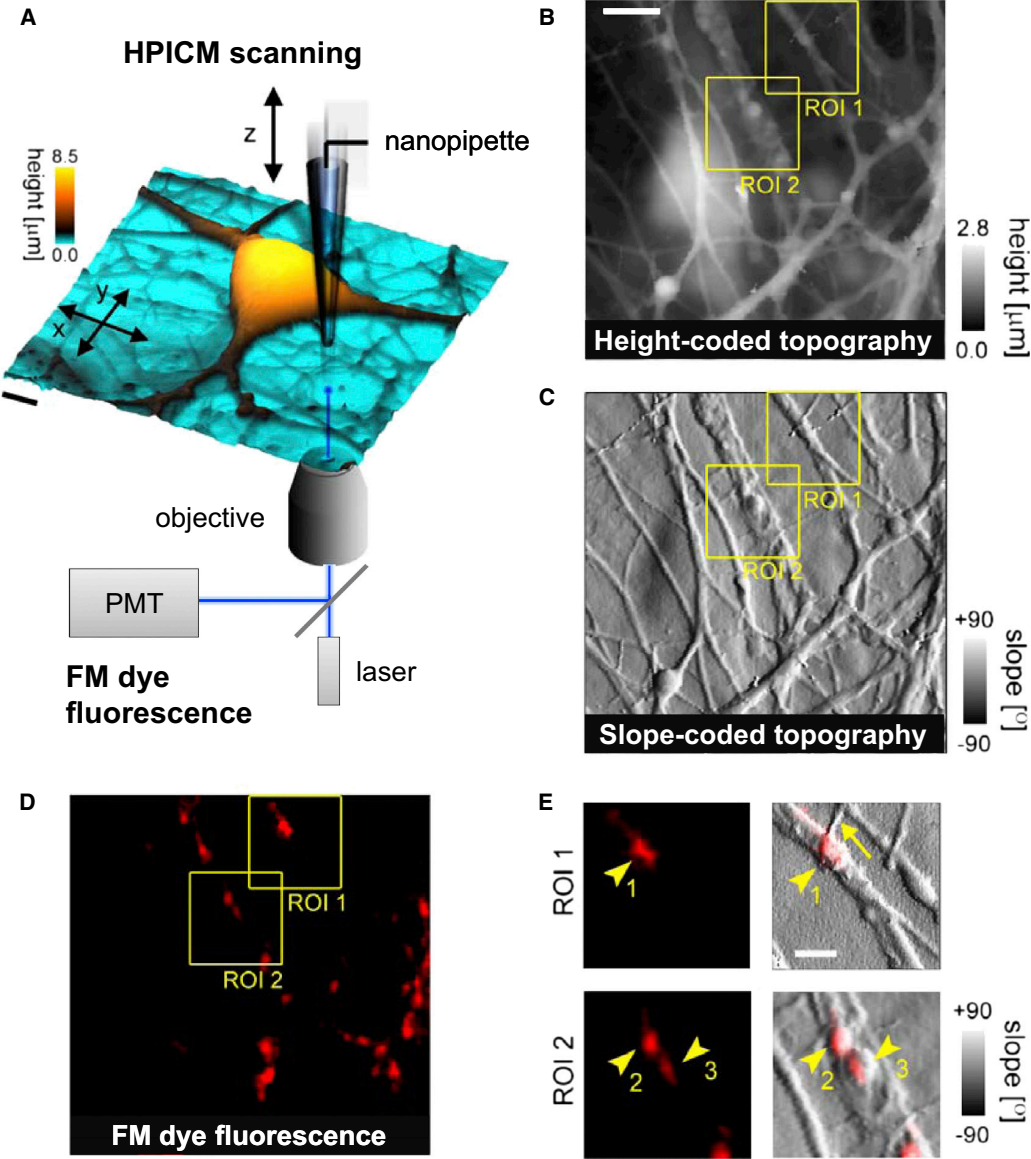
Identification and Superresolution 3D Topographical Imaging of Live Presynaptic Boutons in Neuronal Cultures (A) Schematic of the experimental set up shown with a typical topographical image of a live neuronal hippocampal culture. To allow simultaneous 3D topography and fluorescence imaging, we aligned the HPICM nanopipette with the laser beam of the fluorescent confocal microscope (Gorelik et al., 2002b). Height is shown both as a 3D projection and as the color scale. (B and C) Height-coded (B) and slope-coded (C) topographical images of cultured hippocampal neuronal network growing on the astrocyte feeding layer. In (B), the height is coded as shades of gray. In (C), the gray scale intensity of each pixel was determined by calculating the local slope Φ=arctandz/dx using data from (B), giving the visual appearance of illumination from the right. (D) Fluorescence image of the same area in the FM1-43 channel, showing areas of evoked synaptic vesicle exo-/endocytosis. (E) High-resolution images of ROIs 1 and 2 (yellow squares in B, C, and D). Arrowheads, putative synaptic boutons (1)–(3); arrow, axon. Scale bars represent 5 μm in (A) and (B) and 2 μm in (E).

We aligned the nanopipette tip with an inverted laser-scanning confocal microscope to keep fluorescence and topographical imaging in exact registration (Novak et al., 2009; Shevchuk et al., 2001) (Figure 1A). We labeled active synaptic boutons with FM1-43 by stimulating vesicular exo- and endocytosis using transient depolarization of the neuronal membrane with elevated extracellular [K^+^] (Experimental Procedures). Active synapses were then precisely located by obtaining high-resolution topographic images in areas containing one or more fluorescent puncta (Figures 1B–1E). Matching the tentative bouton structures in topography and fluorescence thus enabled us to identify and monitor live synaptic boutons with a 3D resolution of approximately 100–150 nm (Figure 1E, arrowheads). In many cases, fine axonal processes were also visualized (e.g., Figure 1E, arrow). This approach allowed us to obtain morphometric estimates for live synaptic varicosities lying on dendritic processes (Figure S1 available online). The volume of identified synaptic boutons thus estimated (*V* = 0.14 ± 0.11 μm^3^, mean ± SD, n = 41, Figure S1) was in good agreement with previous estimates obtained by electron microscopy (e.g., Schikorski and Stevens, 1997; *V* = 0.12 ± 0.11 μm^3^).

### Targeting Small Synaptic Boutons for Spatially Resolved Patch-Clamp Recordings

**Figure 2:**
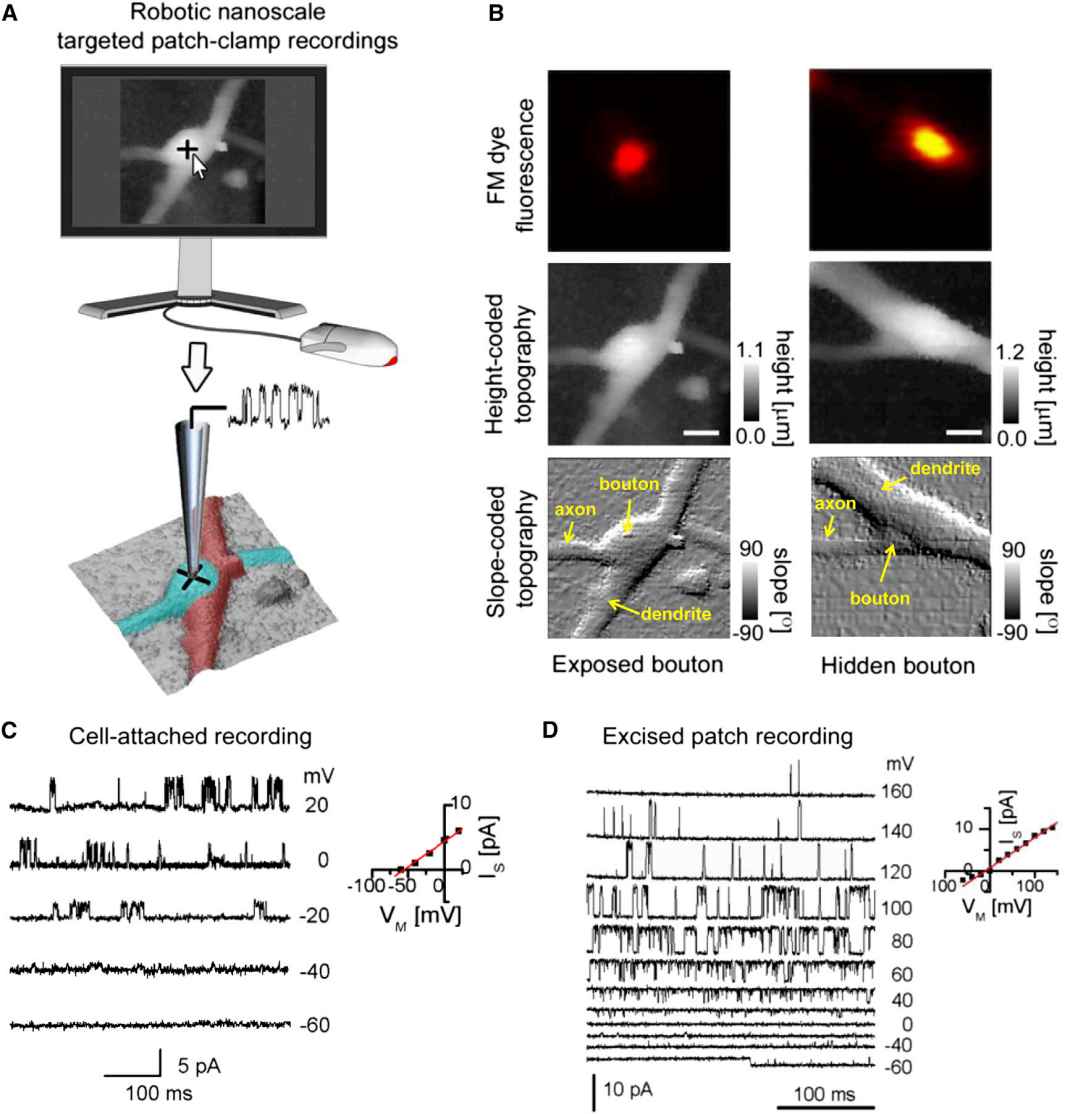
HPICM-Targeted Cell-Attached Patch-Clamp Recordings of Presynaptic Ion Channels (A) Schematic of the experimental set up. Using the recorded 3D topography and an automated computer-controlled algorithm, the scanning pipette was precisely positioned (with nanoscale resolution) onto the synaptic bouton to form a tight seal with the synaptic membrane, allowing targeted single-channel recording. (B) Examples of boutons considered suitable (left) or not suitable (right) for targeted patch-clamp experiments. Only those boutons that were clearly exposed to the pipette (left column, “Exposed bouton”) were selected, while those that were hidden underneath another process (right column, “Hidden bouton”) were rejected (see also Figure S2 for more examples). Top to bottom: FM dye fluorescence; height-coded topography; local slope-coded topography. Scale bar represents 1 μm. (C and D) Examples of single-channel currents recorded from small boutons. (C) A large conductance cation channel, recorded in the cell-attached configuration, with properties consistent with a Ca^2+^-activated K^+^ channel (Sun et al., 1999); current traces (left) and corresponding I/V curve (right). Average slope conductance 78.9 ± 7.0 pS (mean ± SD, n = 6 trials). Voltage values correspond to the derived membrane voltage calculated assuming presynaptic resting membrane potential of −70 mV (Ruiz et al., 2010). (D) Anion-selective putative Cl^−^ channel recorded in the inside-out patch configuration; current traces (left) and corresponding I/V curve (right). Average slope conductance 142.6 ± 14.4 pS (mean ± SD, n = 9 trials). Voltage values correspond to the pipette voltage.

Once an active synaptic terminal suitable for patch-clamp recording had been identified, we used the 3D digital coordinates of the terminal stored in the high-resolution topographic image to move the scanning nanopipette to a selected point on the exposed surface of the terminal and attempted cell-attached single-channel recording (Figure 2A; Experimental Procedures). HPICM was crucial for the selection of boutons suitable for targeted patch-clamp recordings. Indeed, while the FM1-43 fluorescence image allows active boutons to be located in the x-y plane (with diffraction-limited resolution of ∼300 nm in our optical system), it does not provide any information about the relative positions of the pre- and postsynaptic membranes, which are not stained with the FM dye. Thus, the FM1-43 fluorescence image alone does not distinguish between boutons lying above, to one side, or underneath dendrites. In contrast, height-coded HPICM topographical images (in which z coordinates are represented by shades of gray) allow direct identification of the exposed presynaptic boutons. For example, although both regions of interest (ROIs) in Figure 2B contain functional synaptic boutons, the superresolution HPICM images (Figure 2B, bottom) reveal that, while the bouton shown in the left column is exposed to the patch-clamp pipette, the major part of the bouton shown in the right column is hidden under the postsynaptic dendrite. Thus, targeting of small presynaptic boutons using only optical microscopy may lead to nonspecific recording from other structures.

To select boutons for patch-clamp experiments, we applied the following criteria: (1) the presynaptic bouton should be situated on top of, or to the side of, a putative dendritic process, and part of its surface should be accessible to the vertical scanning nanopipette; (2) fine axonal processes connected to the bouton should be detected; and (3) the presynaptic bouton should be clearly distinct from other neuronal structures. More examples of synaptic boutons that satisfy or do not satisfy the above criteria are shown in Figure S2.

### Single-Channel Recordings from the Surface of Active Synaptic Boutons

We first used HPICM-targeting to obtain single-channel recordings from the exposed surface of small synaptic boutons. We achieved the cell-attached patch-clamp configuration with a success rate of 67%. Channels were recorded in 36 out of 46 successful patches (∼78%). Using Monte Carlo simulations (Figure S3), we estimate that, with a 99% confidence interval, the upper limit of the average density of detected channels was in the range of 56–130 channels per μm^2^. We varied the pipette solution systematically in different experiments to provide a preliminary identification of the ion channels detected (Experimental Procedures). When the pipette was filled with the extracellular solution, we observed both low- and high-conductance channels, with reversal potentials consistent with permeability to K^+^. Putative BK channels were identified by a negative reversal potential, a voltage-dependent opening probability, and a large conductance (Figure 2C). The presence of such channels in presynaptic boutons is consistent with immunoelectron microscopy data in small hippocampal synapses and with patch-clamp recordings from large synapses (Hu et al., 2001; Sun et al., 1999). We also made excised inside-out patch recordings from boutons and found channels reversing at ∼0 mV in symmetrical Cl^−^; some of these had a large conductance (Figure 2D) and closed upon depolarization. The properties of these channels were similar to those of anion channels reported in synaptosome recordings (Hosokawa et al., 1994; Nomura and Sokabe, 1991). Although precise identification of all channel types detected is beyond the scope of this study, our findings provide evidence that a variety of ion channels reported previously with indirect methods do occur in live presynaptic axonal boutons.

### Controlled Widening of Scanning Nanopipette Tips to Allow Whole-Cell Presynaptic Recordings

**Figure 3:**
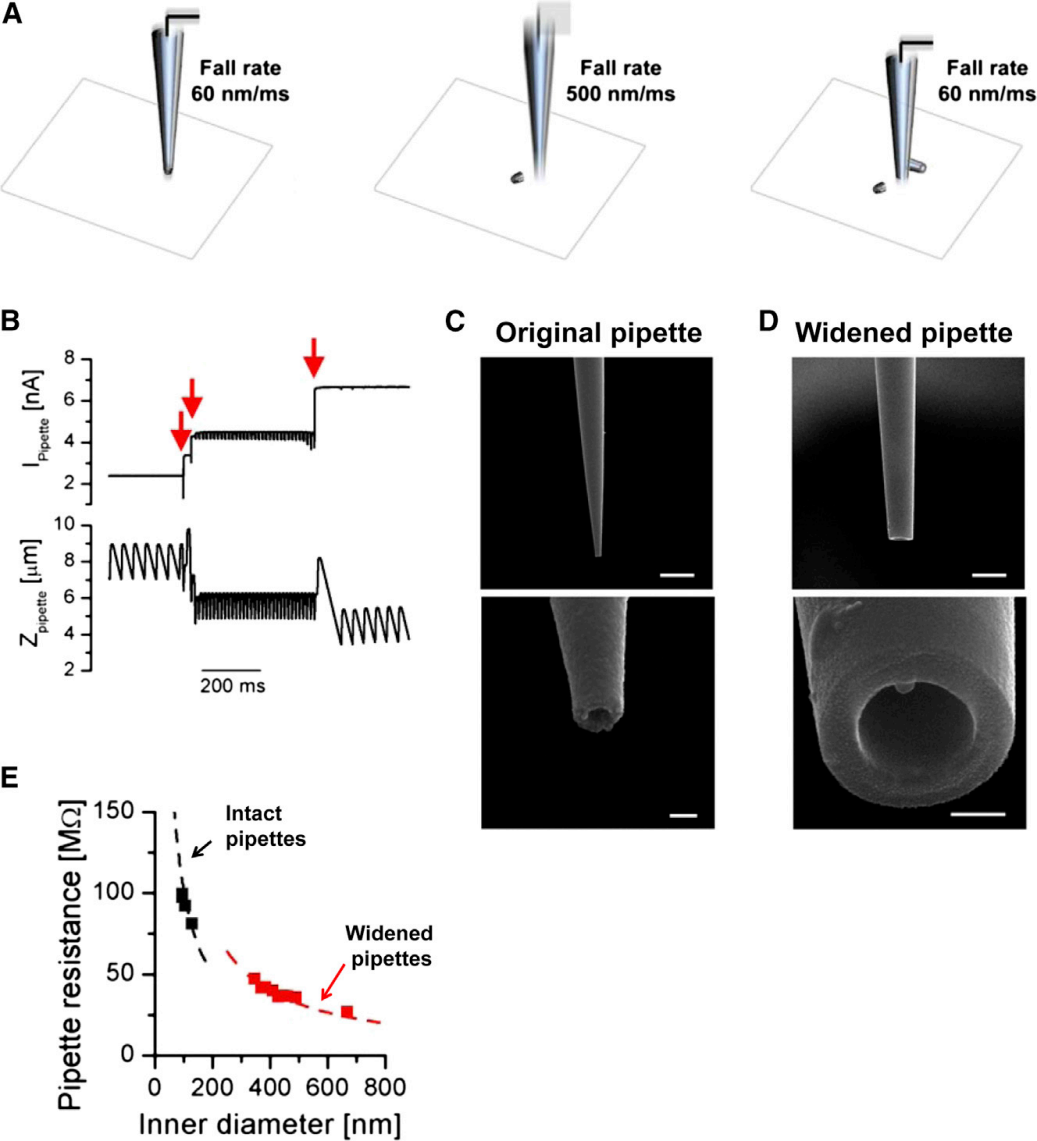
Controlled Pipette Tip Widening (A) Schematic illustrating the pipette widening procedure; see main text for details. (B) Traces of pipette z position and pipette current during the breaking procedure. Every time a small fragment of the pipette tip is chopped off, the pipette current increases (red arrows). The holding pipette voltage was kept constant at 200 mV. (C and D) SEM images of two representative “twin” nanopipettes pulled from the same capillary (Experimental Procedures), one of which was widened using the controlled breaking procedure. Side views of the intact pipette (C) and widened pipette (D) at low (top) and high (bottom) magnification. Scale bars represent 1 μm in top of (C), 100 nm in bottom of (C), 1 μm in top of (D), and 200 nm in bottom of (D). (E) Relationship between pipette resistance and inner tip diameter for intact pipettes (black) and widened pipettes (red). Dashed lines are theoretical predictions calculated using the tip geometry: $R_{pipette}={\left(\pi \cdot d/2\cdot \;\tan\left(\varphi /2\right)\cdot \rho \right)}^{-1}$ (where *d* is the pipette inner tip diameter, φ is the tip cone angle, and ρ ∼1.2 S m^−1^ is the conductivity of the pipette solution [Ying et al., 2002]). Note that because of the ogive cross-section of the pipette, the widening procedure led to a decrease of the tip cone angle from φ = 6.2° ± 0.5° (mean ± SD, n = 4) to φ = 3.8° ± 0.3° (mean ± SD, n = 8).

A major limitation of smart patch clamp is its restriction to the exposed membrane directly accessible to the vertically oriented nanopipette. Ion channels in and near the active zone (AZ) are hidden from the patch pipette, but the currents mediated by these channels could in principle be recorded by rupturing the membrane patch of the terminal to enter the whole-cell patch-clamp configuration. Resolving individual synaptic boutons and axons on 3D topographical images, however, requires high-resistance pipettes with a small inner tip diameter (∼100 nm; Novak et al., 2009; Rheinlaender and Schaffer, 2009; see also below). Therefore, in practice, we could not break the presynaptic cell membrane and obtain whole-cell patch-clamp recordings when using the original scanning nanopipettes. To overcome this limitation, we optimized a method to widen the ultra-fine pipette tip after the completion of the high-resolution 3D topography scan by breaking it against the glass coverslip (Böhle and Benndorf, 1994), using programmable feedback control of the HPICM scanner controller. The nanopipette tip-breaking procedure consisted of three steps (Figure 3A). First, the pipette was navigated to a previously identified area of the coverslip free of neuronal processes. Second, the fall rate (the rate at which the pipette repeatedly approaches the surface during “hopping”) was increased from the standby rate (typically 60 nm/ms) by approximately one order of magnitude (to ∼500 nm/ms). At this fall rate, the noncontact mode of HPICM could no longer be preserved because of the inherent latency of the z axis piezo feedback control. As a result, the pipette repeatedly crashed into the coverslip, breaking its tip and increasing its diameter because of the conical shape of the pipette. Pipette tip breaking resulted in stepwise increases of the pipette current as its resistance dropped (red arrows in Figure 3B). The breaking was automatically stopped by returning the fall rate to baseline (60 nm/ms) once the pipette current reached a desired level. This process could be repeated to fine-tune the desired pipette tip diameter in steps as small as 10% by varying the stop criteria for current increase, duration, and “breaking” fall rate (Figure 3B).

To characterize the properties of widened nanopipettes, we obtained scanning electron microscopy (SEM) images of intact and modified pipette tips (Figures 3C and 3D; see Experimental Procedures for details). Importantly the controlled breaking procedure did not change the overall shape of the pipette tip but reliably allowed the inner tip diameter to be increased approximately 4-fold: from 107 ± 16 nm (mean ± SD, n = 4) to 417 ± 48 nm (mean ± SD, n = 8). The experimentally determined relationship between pipette resistance and inner pipette tip diameter for both intact and widened pipettes was in close agreement with theoretical predictions based on the tip geometry (Figure 3G). On average the resistance of the widened pipettes was decreased ∼2.4-fold (from 92.2 ± 8.9 MΩ to 38.7 ± 4.0 MΩ, mean ± SD, n = 17), thus making the modified pipettes more suitable for whole-cell patch-clamp recordings.

### Targeted Whole-Cell Recordings from Small Boutons

**Figure 4:**
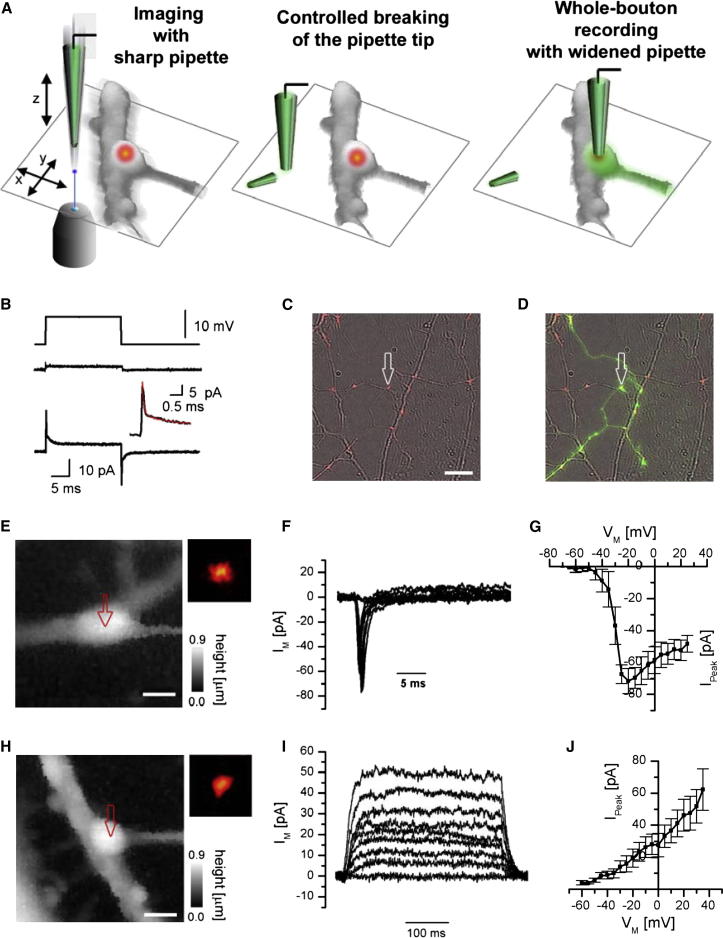
Whole-Cell Patch-Clamp Recordings in Small Synaptic Boutons (A) Principle of the procedure. Left: a high-resolution topographic image was first obtained using a sharp nanopipette containing the green fluorescent morphological tracer Alexa Fluor 488 (200 μM); middle: after identification of a suitable synaptic bouton, the pipette tip was widened (see Figure 3); and right: the modified pipette was used to obtain a whole-bouton recording, allowing diffusion of the Alexa dye into the bouton and nearby axon. (B) Representative passive current responses to a 10 mV square voltage command (top trace) recorded in the bouton-attached configuration (middle trace) and after breaking into the whole-bouton configuration (bottom trace). Insert: double-exponential fit (red) of the capacitive transient. (C) Example showing an overlay of FM-stained synaptic terminals (in red) with bright-field image of neuronal culture prior to whole-cell recording. White arrow marks the bouton where the whole-cell recording was subsequently obtained. (D) The same area as in (C) showing specific labeling of the patched bouton and the adjacent axon with Alexa Fluor 488 (green channel) diffusing from the pipette after establishing the whole-bouton recording (image obtained after pipette withdrawal). (E and F) Example of presynaptic whole-bouton Na^+^ current recordings (see Experimental Procedures for details). (E) Height-coded bouton topography (red arrow, target of location of the whole-bouton recording; insert: corresponding FM-dye fluorescence image. (F) Recorded Na^+^ current traces. (G) Average I/V dependence of whole-bouton Na^+^ currents (mean ± SEM, n = 5). (H and I) Example of presynaptic whole-cell K^+^ current recordings. (H) Topography of the bouton and (I) K^+^ current traces. (J) Average I/V dependence of whole-bouton K^+^ currents (mean ± SEM, n = 5). Scale bars represent 10 μm in (C) and 1 μm in (E) and (H).

Importantly, because the pipette was held vertically at all times, the x, y coordinates of the pipette tip did not change (Figures S4A–S4C). Therefore, the widened pipette tip could be navigated in exactly the same way as described previously for the sharp pipettes, to the presynaptic bouton, where it was applied to the membrane to obtain a gigaseal (Figures 4A and 4B). The pipette-breaking procedure did not interfere with formation of a gigaseal ($R_{seal}$ = 9.4 ± 5.7 GΩ, mean ± S.D., n = 41), which was obtained with a success rate of 57%. Furthermore, the whole procedure of controlled pipette breaking and subsequent formation of a gigaseal at a specific location could be repeated several times (Figure S4D), offering the possibility to perform multiple patch-clamp recordings from different structures using the same pipette.

After establishing a gigaseal with a widened pipette, we ruptured the presynaptic membrane and obtained the whole-bouton patch-clamp recording configuration (Figure 4B, overall success rate 41%). To confirm the identity of the recorded structure, we routinely included the soluble fluorescence tracer Alexa Fluor 488 in the pipette solution and verified that this loaded the patched boutons and adjacent axon (Figures 4C and 4D).

To characterize the basic electrical parameters of the whole-bouton recordings, we used a two-compartment model that was previously utilized to describe presynaptic whole-cell recordings in rod bipolar axonal terminals (Oltedal et al., 2007) (Experimental Procedures). We estimated an upper limit for the access resistance ($R_{A}$ = 156.1 ± 38.2 MΩ, mean ± SD, n = 10) by fitting the capacitive current transients generated by step command voltages using a sum of two exponential functions (Figure 4B). The average time constants of the two exponential components were $\tau_{1}$ = 0.074 ± 0.024 ms and $\tau_{2}$ = 1.3 ± 0.5 ms (mean ± SD, n = 10), which corresponded to capacitances *C_1_* = 0.621 ± 0.226 pF, *C_2_* = 0.962 ± 0.655 pF, and access resistance for the second capacitance $R_{2}$ = 1.6 ± 1.1 GΩ (mean ± SD, n = 10). It should be noted that *C_1_* and *C_2_* are likely to correspond to the compound capacitances of the axonal arbor and possibly the cell soma (Hallermann et al., 2003; Oltedal et al., 2007) as these values were significantly higher than the expected single bouton membrane capacitance *C_bout_*. Indeed, assuming a specific membrane capacitance of 10 fF/μm^2^ and an average bouton surface area of $S_{bout}$ ∼3.23 μm^2^ (Figure S1), we obtain an average estimate of *C_bout_* ∼32.3 fF and a corresponding estimate of the bouton time constant $\tau_{bout}=R_{A}\cdot C_{bout}$ ∼5 μs. Thus, the capacitive transient corresponding to bouton membrane charging could not be properly resolved in the time domain since $\tau_{bout}$ is comparable to the full bandwidth of the patch-clamp amplifier.

The small $\tau_{bout}$, on the other hand, should allow accurate voltage clamping of the bouton compartment despite the high access resistance $R_{A}$. Indeed, using different recording solutions and pharmacological blockers (see Experimental Procedures for details), we obtained whole-bouton recordings of fast Na^+^ currents (Figures 4E–4G, peak current −71.7 ± 16.0 pA, mean ± SD, n = 5 boutons) and slow K^+^ currents (Figures 4H–4J, average current at 0 mV membrane potential 27.6 ± 16.2 pA, mean ± SD, n = 5).

### Presynaptic Voltage-Gated Calcium Channel Recordings

Synaptic release of neurotransmitters is triggered by Ca^2+^ influx via presynaptic voltage-gated calcium channels (VGCCs). VGCCs are enriched inside the AZ (Bucurenciu et al., 2008; Harlow et al., 2001; Holderith et al., 2012; Sheng et al., 2012; Sun et al., 2006), where they are an integral part of the exocytosis machinery (Cao et al., 2004; Han et al., 2011; Kaeser et al., 2011; Mochida et al., 2003), providing for direct coupling between Ca^2+^ entry and neurotransmitter release. Accumulating data argue that VGCC activity is regulated at the level of individual small presynaptic boutons and that this mechanism contributes to target-specific adjustment of presynaptic strength (Ermolyuk et al., 2012; Holderith et al., 2012; Koester and Johnston, 2005). However, until now direct electrophysiological recordings of presynaptic VGCCs were only possible in large synapses such as the calyx of Held or hippocampal mossy fiber bouton (Bischofberger et al., 2006; Schneggenburger and Forsythe, 2006).

**Figure 5:**
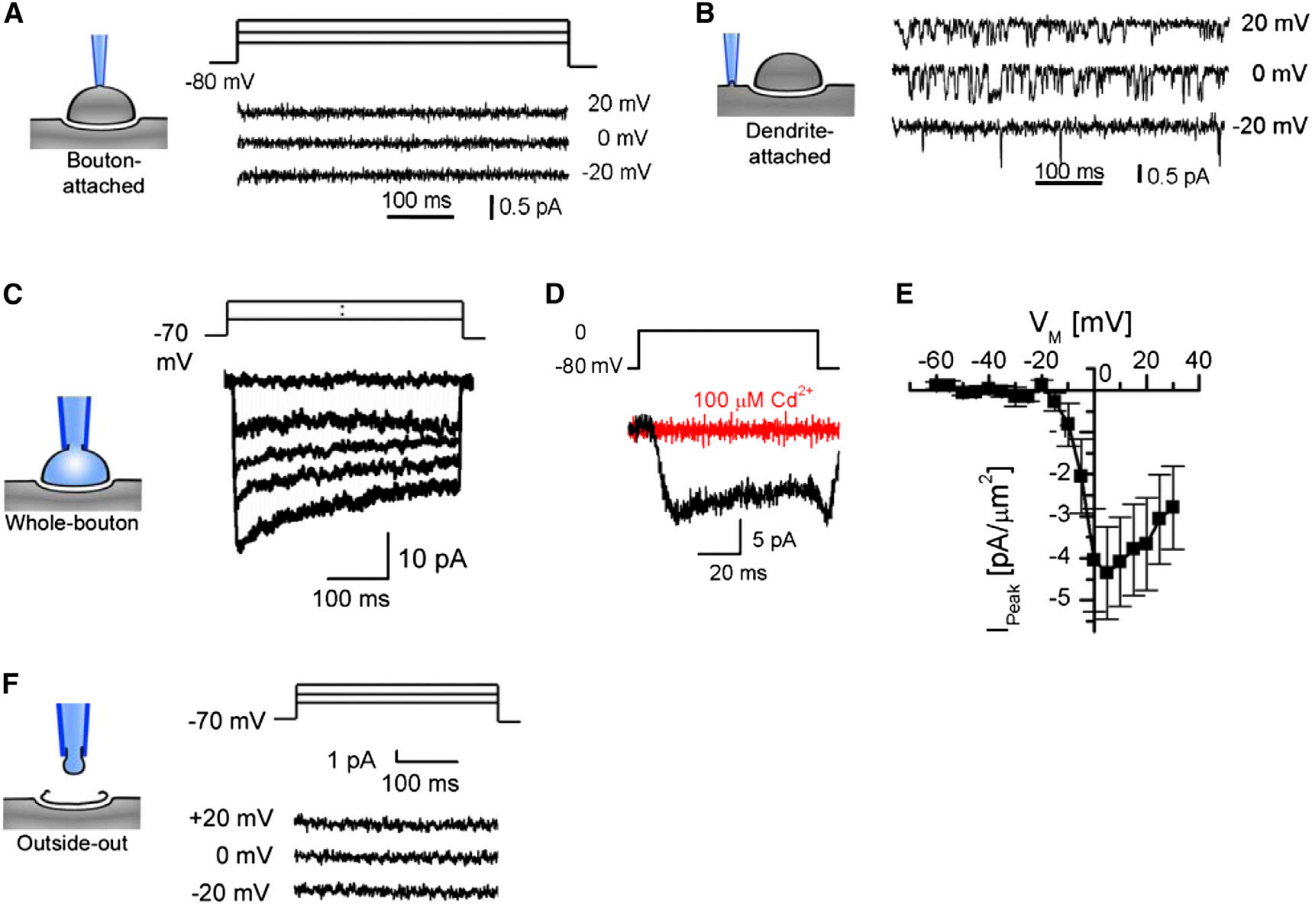
Presynaptic VGCC Recordings (A) Representative cell-attached recording made on the exposed surface of a presynaptic bouton. No VGCC activity was found in n = 65 cell-attached patches. (B) Example of cell-attached recordings of VGCC activity in dendrites. (C–E) Whole-cell recordings of VGCC activity in small synaptic boutons. (C) Representative recording of Ca^2+^ current traces corresponding to different voltage step commands. (D) Example of whole-cell Ca^2+^ current block by extracellular application of 0.1 mM CdCl_2_. (E) Average I/V dependence of whole-bouton Ca^2+^ currents normalized to the bouton surface area (mean ± SEM, n = 6). (F) Absence of VGCC activity in the outside-out recording obtained after successful whole-bouton recordings of VGCC activity.

To assess properties of VGCCs in small presynaptic boutons, we first attempted HPICM-targeted cell-attached recordings from the exposed bouton surface. To optimize the recording conditions for the detection of VGCCs, we used a Ba^2+^-containing pipette solution and switched the bath to a high K^+^ extracellular solution to collapse the resting membrane potential of neurons (Delmas et al., 2000) (Experimental Procedures). Strikingly, we found no evidence for VGCCs in 44 bouton patches with unmodified scanning nanopipettes and in 21 patches with widened (broken) pipettes at different parts of the exposed surface of small boutons (e.g., Figure 5A). Twelve of these patches nevertheless contained identifiable anion channels (data not shown). We estimate that the density of VGCCs on the exposed surface of axonal boutons was less than six channels per bouton (σ < 3.9 channels per μm^2^, Monte Carlo simulations with 99% confidence interval; Figure S3). Despite the absence of detectable VGCCs on the exposed surface of boutons, we readily recorded Ca^2+^ channels in postsynaptic dendrites (in 2 out of 17 patches, corresponding to an estimated upper limit of the average channel density between 2 and 21 channels per μm^2^; Figure 5B and Figure S3). The apparent absence of VGCCs on the exposed surface of presynaptic boutons is in full agreement with recent findings that the overwhelming majority of functional VGCCs in central synapses are located in the AZ (Bucurenciu et al., 2008; Holderith et al., 2012; Sheng et al., 2012).

In contrast to the cell-attached recordings, we readily detected VGCC activity in whole-bouton recordings (when conditions were optimized for VGCC detection, see Experimental Procedures), with access to the whole membrane of a presynaptic bouton including the AZ (Figures 5C–5E). The average value of peak Ca^2+^ current in the whole-bouton mode normalized by the presynaptic bouton surface area (directly obtained from the high-resolution HPICM bouton images, see Figure S1 for details) was –4.4 ± 2.7 pA/μm^2^ (mean ± SD, n = 6). In some experiments (n = 3), we also verified that the recorded currents were completely abolished by extracellular application of the nonspecific VGCC blocker Cd^2+^ (Figure 5D). Interestingly, we failed to detect VGCCs in the bouton membrane remaining in the outside-out patches (Figure 5F), obtained by slowly withdrawing the pipette away from the bouton upon completion of the whole-bouton recording (n = 3), even though Ca^2+^ currents were recorded in whole-bouton mode. Because the AZ in these experiments is likely to remain firmly attached to the postsynaptic density (Berninghausen et al., 2007), and is therefore inaccessible to the outside-out configuration, this result further confirms that the majority of VGCCs in small central synapses are concentrated within the AZ.

## Discussion

The combination of topographical imaging, nanopositioning, and controlled pipette tip breaking described here allowed us to overcome the optical limit in spatial resolution of conventional patch-clamp techniques and to obtain targeted cell-attached and whole-cell recordings from small presynaptic boutons with a characteristic size of ∼1 μm.

The method described here is limited to neurons in culture where exposed synaptic terminals are directly accessible to scanning nanopipettes. Importantly, synapses in cultured neurons retain most of the functional and morphological properties of synapses in the brain (Schikorski and Stevens, 1997) and are therefore widely used as a “first choice” model system when elucidating the basic cellular and molecular mechanisms of transmitter release and homeostatic synaptic plasticity. Outside of cultures, our current quantitative understanding of presynaptic ion channel function relies mostly on studies at large synapses such as the Calyx of Held or hippocampal mossy fiber boutons, which are amenable to direct patch-clamp recordings. However, presynaptic signaling in these large specialized synapses differ in several respects from that in small central synapses (P. Jonas and N.P. Vyleta, 2012, SFN, abstract; Schneggenburger and Forsythe, 2006). Therefore, the set of techniques described here should provide novel and important insights into the presynaptic physiology of small central synapses. Importantly, the integration of HPICM components into an electrophysiological laboratory is relatively straightforward, especially in comparison with other scanning probe microscopy techniques, and can be performed as an “upgrade” of virtually any existing patch-clamp set up based on an inverted microscope.

Among questions that lend themselves to these methods are: what are the expression levels and biophysical properties of different ion channels found in small synaptic boutons? How are these channels distributed among individual synapses? How does the identity of presynaptic ion channels regulate the AP waveform, presynaptic Ca^2+^ dynamics, and synaptic vesicle exo-/endocytosis? Although the absolute values of capacitance of small presynaptic boutons may not be resolvable by the time domain method, changes of the bouton capacitance during synaptic vesicle exo-/endocytosis may in principle be detectable when using high-frequency sine wave stimulation and lock-in detection, as previously reported in larger mossy fiber boutons and rod bipolar cells (Hallermann et al., 2003; Oltedal et al., 2007). Furthermore, direct access to presynaptic boutons via the patch pipette should not only allow one to control the presynaptic membrane potential but also to measure and manipulate the presynaptic Ca^2+^ concentration by direct loading of synthetic Ca^2+^ dyes and Ca^2+^-caging compounds. Until now, these types of experiments were only possible in large synapses such as the calyx of Held. In summary, we anticipate that the combined application of HPICM-assisted patch-clamp recordings, together with previously described electrophysiological and imaging methods to image vesicular release and Ca^2+^-dynamics in individual synaptic boutons (e.g., Ariel and Ryan, 2010; Ermolyuk et al., 2012; Hoppa et al., 2012; Li et al., 2011; Li and Tsien, 2012), will provide answers to these and other questions relating to the behavior of small central synapses.

## Experimental Procedures

### Cell Cultures and Recording Solutions

Hippocampal neurons were isolated from P1–P2 rat pups and cultured in Neurobasal-based medium either on an astrocyte feeder layer or on poly-D-lysine-treated coverslips. All recordings were conducted at ambient temperature (23°C–26°C) 12–19 days after plating. The standard extracellular solution used in all experiments contained 125 mM NaCl, 2.5 mM KCl, 2 mM MgCl_2_, 2 mM CaCl_2_, 30 mM glucose, 0.01 mM NBQX, 0.05 mM APV, and 25 mM HEPES (pH 7.4). Active synapses were labeled with 20 μM (bath concentration) FM1-43 (Invitrogen) or 200 μM SynaptoRed C1 (SRC1, Biotium) by incubation in the extracellular solution, with 90 mM NaCl replaced by 90 mM KCl for 90 s followed by a 10–15 min wash in the original solution. Tetrodotoxin (1 μM) was added to the extracellular solution in some experiments to slow down spontaneous destaining of the FM dyes.

### High-Resolution Scanning and Identification of Active Boutons

HPICM topographic images were obtained using a custom-modified SICM sample scanner ICNano-S (Ionscope) and custom software as described previously (Novak et al., 2009). Briefly, the scan head consisted of a PIHera P-621.2 X-Y Nanopositioning Stage (Physik Instrumente [PI]) with 100 × 100 μm travel range that moved the sample and a LISA piezo actuator P-753.21C (PI) with travel range 25 μm for pipette positioning along the z axis. Coarse sample positioning was achieved with translation stages M-111.2DG (x-y directions) and M-112.1DG (z axis) (PI). The z piezo actuator was driven by a 200 W peak power high-voltage PZT amplifier E-505 (PI), while the x-y nanopositioning stage was driven by 3 × 14 W amplifier E-503 (PI). All piezo elements operated in capacitive sensor-controlled closed loop using Sensor & Position Servo-Control Module E-509 (PI). The scan head was placed onto an inverted Nikon TE2000-U microscope (Nikon) table equipped with differential micrometers (OptoSigma) for precise positioning. A custom-built laser confocal set up was used to record fluorescence simultaneously with topography. Excitation was provided by an LCS-DTL-364 laser diode (473 nm wavelength, Laser Compact). The fluorescence signal was collected using a 100× 1.3 NA oil-immersion objective, an epifluorescence filter block, and a photomultiplier with a pinhole (D-104-814, Photon Technology International) or in nonconfocal mode using wide-field illumination and an Evolve 512 EM-CCD camera (Photometrics).

Fine-tipped nanopipettes used both to probe the neuronal topography and to perform cell-attached patch-clamp recordings were pulled from borosilicate glass (OD 1 mm, ID 0.5 mm, Sutter Instruments) using a horizontal laser-based puller P-2000 (Sutter Instruments). The pipette resistance was in the range of ∼80–110 MΩ, corresponding to an estimated inner tip diameter of ∼90–125 nm (Figure 3E).

Nanopipettes were held in voltage-clamp mode with an Axopatch 200B patch-clamp amplifier coupled to a DigiData 1322A interface (Molecular Devices). Topographic and confocal images were obtained, first, by aligning the nanopipette tip with the fluorescence microscope focal plane and, second, by recording topographic and fluorescence images while scanning the specimen in the x and y axes by the SICM electronics.

### Scanning-Electron Microscopy of Pipette Tips

The laser-pulling process generates, from a single capillary, a pair of “twin” nanopipettes with virtually identical geometries. One of the pair was used as a representative of the tip geometry before pipette breaking. The other was subjected to the controlled widening procedure as described in the main text. In this set of experiments, the ultrafiltered standard extracellular solution (20 nm syringe filter) was used both in the pipette and in the bath. The pipette resistance was monitored before and after the breaking procedure using the Seal Test function of pCLAMP 9.2 (Molecular Devices). Immediately after completion of the breaking procedure, the pipette solution was removed and the pipette tip was washed three times with ultra filtered 96% ethanol and dried. Both modified and unmodified pipettes were sputter coated with gold (15 nm coat thickness) and imaged using an FEI Quanta 3D FEG (FEI) scanning electron microscope operating in high vacuum mode at 30 kV. Dimensions and cone angle of pipette tips were measured in ImageJ (U.S. National Institutes of Health).

### Single-Channel Cell-Attached and -Excised Patch-Clamp Recordings

After topographic and confocal images were obtained, the coordinates of a defined ROI on the neuron surface (synaptic bouton or dendrite) were used for precise positioning of the SICM pipette for cell-attached patch-clamp recording. The nanopipette was then lowered by the z axis piezo control until it made contact with the cell surface. Light suction was used to form a gigaohm seal (previously reported as “smart patch clamp” [Gorelik et al., 2002a; Gu et al., 2002]). Single-channel currents were filtered at 1 kHz and sampled at 20 kHz. Data acquisition and analysis were done using pCLAMP 9.2 (Molecular Devices).

Cell-attached and excised patch recordings in Figures 2C and 2D were performed using the same standard extracellular solution in the bath and in the scan pipettes. To investigate Ca^2+^ channels (Figures 5A and 5B), we used a pipette solution that contained 90 mM BaCl_2_, 10 mM HEPES, 10 mM TEA-Cl, 3 mM 4-aminopyridine, adjusted to pH 7.4 with TEA-OH and zeroed cell membrane potential by switching the bath solution after obtaining a gigaseal to 120 mM KCl, 3 mM MgCl_2_, 5 mM EGTA, 11 mM glucose, and 10 mM HEPES (pH 7.4) as described previously (Delmas et al., 2000).

### Whole-Cell and Outside-Out Recordings in Small Boutons Using a Broken Pipette

The pipette resistance of widened pipettes used for whole-cell recordings in small synaptic boutons was within the range 35 to 45 MΩ, corresponding to an inner tip diameter of ∼350–450 nm (Figure 3E). Once a gigaseal was formed, suction pulses were used to break the membrane patch to obtain the whole-cell configuration. Electrical parameters of whole-bouton recordings were assessed with a two-compartment model of passive membrane properties previously used in axon terminals of rod bipolar cells (Oltedal et al., 2007). Briefly, the capacitive current transients were fitted using a sum of two exponential functions $I\left(t\right)=A_{1}\;\exp\left(-t/\tau_{1}\right)+A_{2}\;\exp\left(-t/\tau_{2}\right)+I_{s}$, and the access resistances and the capacitances for both compartments were calculated using Equations (3)–(6) from (Oltedal et al., 2007). The membrane capacitance in whole-cell recordings was not actively compensated and the specific ion-channel currents free of capacitive transients were obtained using a P/N leak subtraction protocol implemented in the pCLAMP 9.2 acquisition software. Whole-bouton Na^+^ current recordings (Figures 4E–4G) were performed using the standard extracellular solution without Ca^2+^ in the bath and a pipette solution containing 135 mM CsMeSO_4_, 2 mM MgCl_2_, and 10 mM EGTA (pH 7.4 with CsOH). Whole-cell K^+^ current recordings (Figures 4H–4J) were performed with a Ca^2+^-free extracellular solution containing 1 μM tetrodotoxin and a pipette solution containing 135 mM KMeSO_4_, 10 mM HEPES, 10 mM Na-Phosphocreatine, 4 mM MgCl_2_, 4 mM Na_2_ATP, and 0.4 mM Na_2_GTP. Whole-bouton Ca^2+^ current recordings (Figures 5C–5E) were performed in the standard extracellular solution (containing 2 mM CaCl_2_) supplemented with 1 μM tetrodotoxin. The pipette solution contained 145 mM CsMeSO_4_, 2 mM MgCl_2_, 2 mM Na_2_ATP, 0.3 mM Na_2_GTP, 10 mM HEPES, 10 mM EGTA, and 5 mM Na-creatine phosphate (pH 7.4 with CsOH). To confirm that recorded Ca^2+^ currents were mediated by VGCCs in some experiments, we added 0.1 mM CdCl_2_ to the extracellular solution. In outside-out experiments (Figure 5F), the extracellular solution was replaced by buffer containing 135 mM CsGluconate, 20 mM BaCl_2_, and 10 mM HEPES (pH 7.4 with CsOH).

## Author Contributions

The manuscript was written by P.N. and K.E.V. All authors discussed the results and commented on the manuscript. Y.E.K., K.E.V., and G.W.M conceived and designed the project. P.N., J.G., A.I.S., U.V., R.J.B., and Y.S.E. performed the experiments.
