# Supplementary figures and images for: Nanoscale-Targeted Patch-Clamp Recordings of Functional Presynaptic Ion Channels
Source: Neuron. 2013 Sep 18;79(6):1067–77. doi: 10.1016/j.neuron.2013.07.012 (PMC3781326; doi:10.1016/j.neuron.2013.07.012)

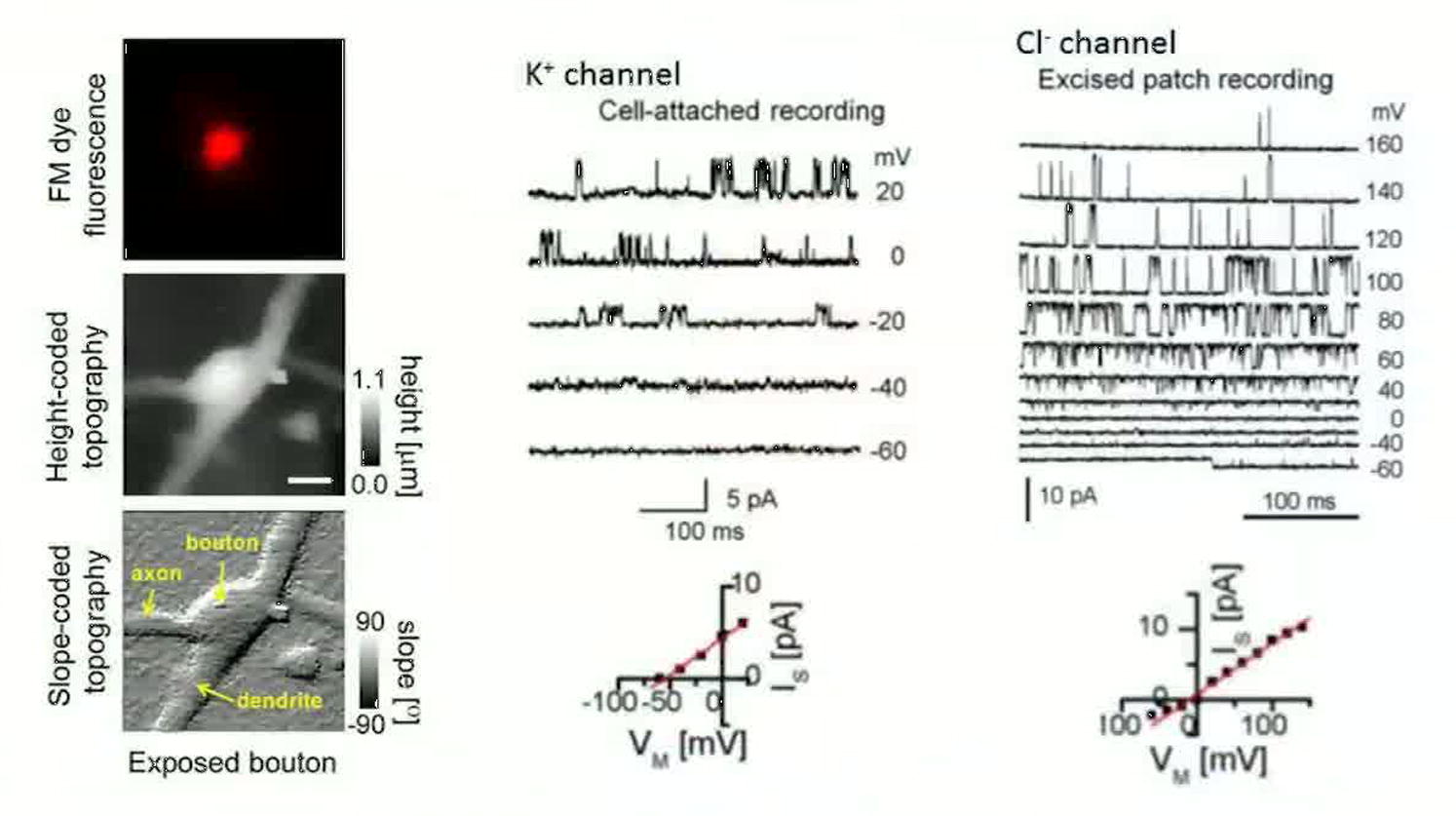

Supplement: Supplementary file 1 [file mmc2.jpg]
