# Supplementary material for: Nanoscale-Targeted Patch-Clamp Recordings of Functional Presynaptic Ion Channels
Source: Neuron. 2013 Sep 18;79(6):1067–77. doi: 10.1016/j.neuron.2013.07.012 (PMC3781326; doi:10.1016/j.neuron.2013.07.012)
Supplement: Document S1. Figures S1–S4 [file mmc1.pdf]

Neuron, Volume *79*

Supplemental Information

Nanoscale-Targeted Patch-Clamp Recordings of Functional Presynaptic Ion Channels

Pavel Novak, Julia Gorelik, Umesh Vivekananda, Andrew I. Shevchuk, Yaroslav S. Ermolyuk, Russell J. Bailey, Andrew J. Bushby, Guy W.J. Moss, Dmitri A. Rusakov, David Klenerman, Dimitri M. Kullmann, Kirill E. Volynski, and Yuri E. Korchev

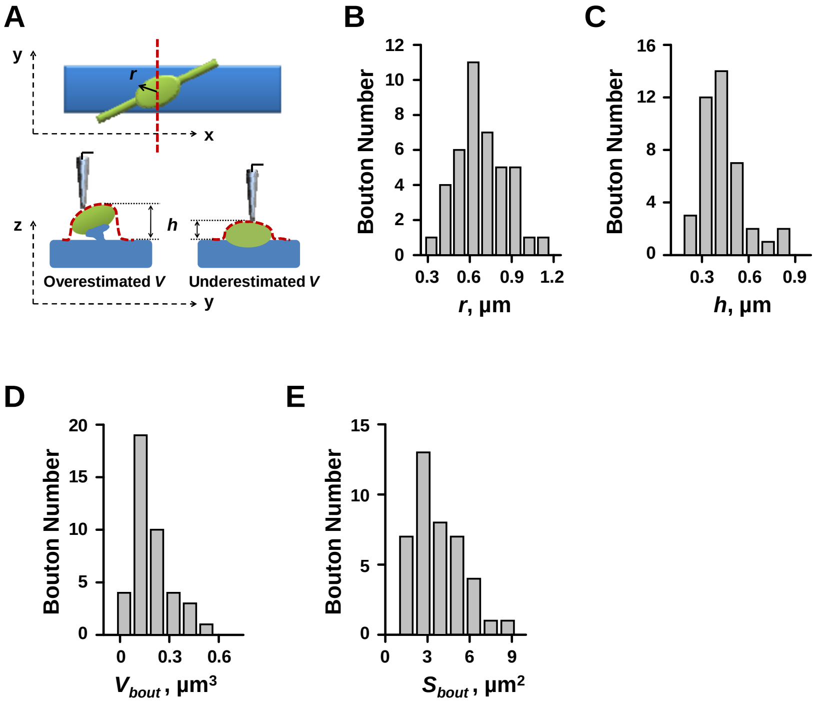

Figure S1 related to Figure 1. Morphometric analysis of live presynaptic boutons in cultured hippocampal neurons. (A) Schematic illustrating measurements of presynaptic bouton size and geometry. (Top) We first measured the apparent area of the bouton projection onto the X-Y plane (S_{proj}) and then calculated the characteristic bouton radius (r) from $r = \sqrt{S_{proj} / \pi}$. (Bottom) We next measured the effective height of the bouton (h) using Y-Z plane projections and estimated the bouton volume (V_{bout}) by direct integration of planar scans over X, Y, and Z (Korchev et al., 2000). Finally we estimated the bouton surface area using an approximate formula for the ellipsoid surface area $S_{bout} = 4\pi[(a^p b^p + a^p c^p + b^p c^p)/3]^{1/p}$, where p = 1.6075. Note that, because of differences in synaptic organization, SICM may either overestimate (left) or underestimate (right) the true value of h , V_{bout} , and S_{bout} .

(B-E) Distributions of the characteristic radii r = 0.63 ± 0.18 μm, (mean ± SD) (B), maximal height h = 0.38 ± 0.14 μm (C), bouton volume V_{bout} = 0.135 ± 0.11 μm^3 (D), and bouton surface area S_{bout} = 3.23 ± 1.71 μm^2 (E) measured for 41 boutons.

Supplemental Reference: Korchev,Y.E., Gorelik,J., Lab,M.J., Sviderskaya,E.V., Johnston,C.L., Coombes,C.R., Vodyanoy,I., and Edwards,C.R. (2000). Cell volume measurement using scanning ion conductance microscopy. Biophys. J. 78, 451-457.

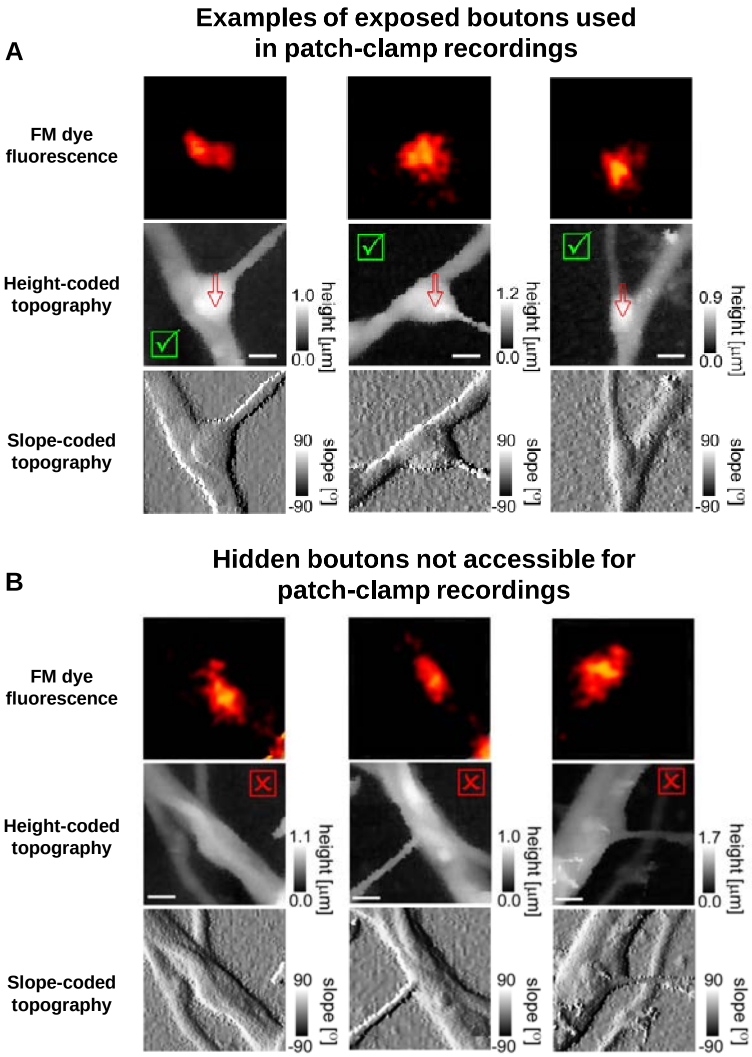

Figure S2 related to Figure 2. Selecting boutons for smart patch clamp recordings.

To select boutons for patch clamp experiments we applied the following criteria: (i) the presynaptic bouton should be situated on top of or to the side of a putative dendritic process and part of its surface should be accessible to the vertical scanning nanopipette, (ii) fine axonal processes connected to the bouton should be detected, (iii) the presynaptic bouton should be clearly distinct from other neuronal structures. (A) Examples of synaptic boutons that satisfy the above criteria which were used in targeted patch clamp recordings. FM1-43 fluorescence (top row), height-coded topography (middle row) and slope-coded topography (bottom row) of well exposed synaptic boutons (red arrows). (B) Examples of boutons rejected because other processes obstruct access by the pipette. Scale bars 1 µm.

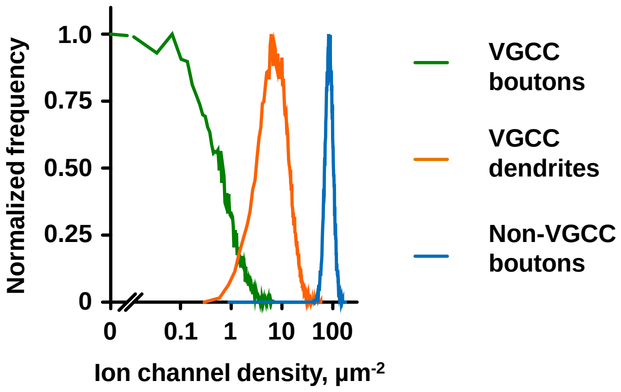

Figure S3 related to Figures 2 and 5. Statistical analysis of patch clamp ion channel recordings from the surface of synaptic boutons and dendrites.

To obtain confidence estimates for the channel density estimates we performed Monte-Carlo simulations replicating our experimental design. Assuming that the available channels are randomly scattered on the exposed cell surface with an unknown density σ, the probability of finding no channels in an individual patch is, according to Poisson statistics, $p(0) = e^{-s\sigma}$, where s is the membrane area sampled by the nanopipette. Using experimental determined average nanopipette tip inner diameter d = 107 ± 16 nm (see main text), we obtained a lower estimate for the membrane area under the patch pipette assuming a hemispherical shape for the membrane patch $S_{patch} = 2\pi(d/2)^2$ ~ 0.018 µm^2. In the simulations, we screened a range of values of σ by randomly selecting them between 0 and 200 channels/µm^2, and for each such value we generated *n* trials (*n* equals the number of real patch clamp experiments) documenting the number of successes (when at least one channel is present). Once this procedure was repeated 10^6 times, we selected only the trials that matched the number of successful patches in each type of real experiments and used those to plot the distributions of channel densities. This allowed us to determine 99% confidence intervals for the corresponding σ (µm^{-2}): 56<σ<130 for overall ion channel density on the exposed surface of the boutons (blue, 36 out of 46 patches), σ<3.9 for the number of VGCCs on the exposed surface of the boutons (green, 0 out of 65 patches), and 1.6<σ<29.1 for VGCCs on dendrites (brown, 2 out of 17 patches). It should be noted that the real membrane patch area is likely to be several fold larger than the hemispherical shape low limit estimate used in the above simulations (Sakmann and Neher, 1995). Therefore the obtained σ values represent upper limit estimates of the real surface channel density.

Supplemental Reference: Sakmann,B., and Neher,E. (1995). Geometric Parameters of Pipettes and Membrane Patches. In Single-Channel Recordings, B. Sakmann, and E. Neher, eds. Plenum Press New York, pp. 637-650.

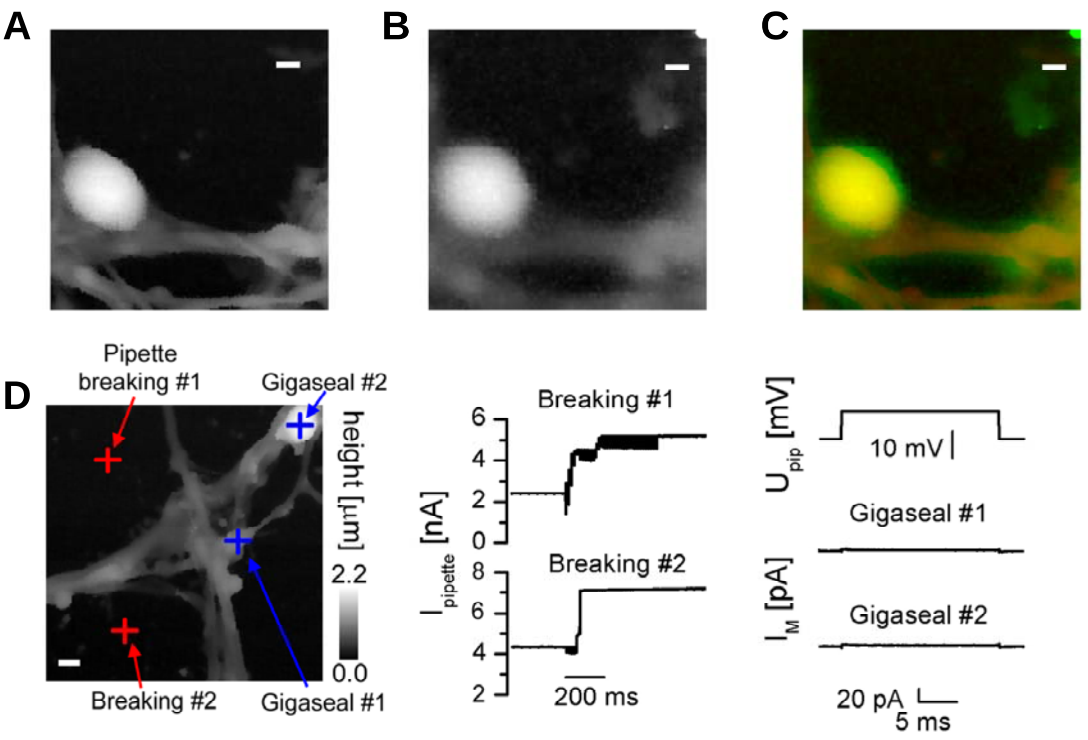

Figure S4 related to Figure 4. HPICM-controlled pipette widening procedure does not change the X,Y coordinates of the pipette tip and does not prevent gigaseal formation.

(A) Topography scanned with sharp pipette before widening procedure. (B) Topography scanned with widened pipette. (C) Overlay of (A) and (B), with (A) shown in red color scale and (B) in green color scale. Note the general loss of lateral resolution in (B) resulting from wider pipette tip diameter. Importantly, because the pipette was held vertically at all times, the X,Y coordinates of the pipette tip did not change during the breaking procedure, as witnessed by the complete alignment of images in (A) and (B).

(D) Pipette breaking procedure does not prevent formation of a gigaseal on the neuronal membrane. After obtaining high-resolution topographical image (left), the original nanopipete was first widened by breaking against the glass coverslip at the location marked "Pipette breaking #1" and a cell-attached gigaseal was established at location marked "Gigaseal #1". The pipette was then slowly withdrawn and the whole procedure was repeated at two different locations ("Pipette breaking #2" and "Gigaseal #2"). Traces of pipette current $I_{pipette}$ during the two breaking procedures are shown in the middle. Current responses to a 10 mV voltage pulse, demonstrating that the resistances of both cell-attached seals were ~ 10 GΩ are shown to the right. Scale bars 1 µm.
